# Correlates of sedentary behaviours in preschool children: a review

**DOI:** 10.1186/1479-5868-7-66

**Published:** 2010-09-08

**Authors:** Trina Hinkley, Jo Salmon, Anthony D Okely, Stewart G Trost

**Affiliations:** 1 Centre for Physical Activity and Nutrition Research, Deakin University, 221 Burwood Hwy, Burwood, Vic, 3125 Australia; 2 Interdisciplinary Educational Research Institute, University of Wollongong, Northfields Ave, NSW, 2522 Australia; 3 College of Health & Human Sciences, Oregon State University, 123 Women's Building, Corvallis, OR 97331, USA

## Abstract

**Background:**

Sedentary behaviour has been linked with a number of health outcomes. Preschool-aged children spend significant proportions of their day engaged in sedentary behaviours. Research into the correlates of sedentary behaviours in the preschool population is an emerging field, with most research being published since 2002. Reviews on correlates of sedentary behaviours which include preschool children have previously been published; however, none have reported results specific to the preschool population. This paper reviews articles reporting on correlates of sedentary behaviour in preschool children published between 1993 and 2009.

**Methods:**

A literature search was undertaken to identify articles which examined correlates of sedentary behaviours in preschool children. Articles were retrieved and evaluated in 2008 and 2009.

**Results:**

Twenty-nine studies were identified which met the inclusion criteria. From those studies, 63 potential correlates were identified. Television viewing was the most commonly examined sedentary behaviour. Findings from the review suggest that child's sex was not associated with television viewing and had an indeterminate association with sedentary behaviour as measured by accelerometry. Age, body mass index, parental education and race had an indeterminate association with television viewing, and outdoor playtime had no association with television viewing. The remaining 57 potential correlates had been investigated too infrequently to be able to draw robust conclusions about associations.

**Conclusions:**

The correlates of preschool children's sedentary behaviours are multi-dimensional and not well established. Further research is required to provide a more comprehensive understanding of the influences on preschool children's sedentary behaviours to better inform the development of interventions.

## Background

Sedentary behaviours typically require low levels of energy expenditure, defined as 1.5 METs or fewer, to perform [1]. Such behaviours generally include television viewing, electronic game use, reading, and computer use.

Sedentary behaviour (predominantly in the form of television viewing) has been shown to be associated with a number of health outcomes, even in preschool-aged (roughly 3-5 years) children. It has been positively associated with adiposity [2-4], and inversely associated with bone mineral content [5]. Cognitive and behavioural outcomes have also been inversely associated with television viewing [6-8], and a meta-analysis [9] showed that exposure to television violence was positively related to more aggressive and anti-social behaviour in young people, with the greatest effect occurring among young children (birth to 5 years).

Sedentary behaviours have shown a moderate tendency to track over time from quite a young age (e.g., from the preschool years) [10,11], particularly in boys [12], and therefore developing strategies that target reduced time spent being sedentary during the period when those behaviours are being established may be beneficial for future health outcomes.

Inadequate data exists on the current prevalence of preschool children's levels of sedentary behaviour, and various measurement and analytic issues hamper comparison of findings between studies. Measurement of sedentary behaviour primarily utilizes self- or proxy-report surveys or log books to capture specific behaviours such as those named above. In addition, objective instruments such as accelerometers or heart rate monitors are used, where sedentary behaviour is defined as being below a given threshold of movement counts or beats per minute, respectively. Most studies using objective methods (such as accelerometry) to assess time in sedentary behaviour report that children spend between 50% and 80% of their time being sedentary [11,13-16]; however, estimates range from 34% [17] to 94.5% [18]. Further, studies using parental proxy-report estimate that preschool age children spend between 1.8 [19] and 3.3 [20] hours per day watching television. Therefore, preschool children spend significant proportions of their waking time being sedentary, and may be at risk of undesirable health outcomes as a result.

As preschool children spend such a large proportion of their time being sedentary, it is important to examine the factors which influence those behaviours. Identification of modifiable correlates will allow researchers to target those correlates when developing interventions to reduce time spent being sedentary. Research into correlates of sedentary behaviour is an emerging field, with the majority of studies examining associations in the preschool population (ages three to five years) being reported since 2002. Reviews of the correlates of sedentary behaviour [21] and television viewing [22] in youth have previously been published; however, none have reported outcomes for preschool children separately from other age groups.

A recent review of correlates of sedentary behaviour in children and adolescents failed to produce sufficient evidence of overall associations in preschool children due to the small number of studies (three) identified which investigated such associations [21]. Previously, Gorely et al. [22] identified 10 studies which investigated correlates of one sedentary behaviour, television viewing, in children from birth to six years, but did not report on those results separately from results for children and adolescents up to 18 years of age. Both those reviews used a social ecological perspective [23] to categorize potential correlates of sedentary behaviour across five levels: (1) demographic and biological; (2) psychological, cognitive, and emotional; (3) behavioural attributes and skills; (4) social and cultural; and (5) physical environmental. While Gorely et al. [22] identified variables across all five levels, consistency between studies was lacking. A review of correlates of physical activity in preschool children [24] identified unique correlates for that population, not identical to those for older children's physical activity. Preschool children are likely to experience different influences on their sedentary behaviours compared with older children who may be influenced by school, peer and broader potential correlates than preschool children. Therefore, it is not possible to postulate that correlates of sedentary behaviour will be the same for preschool children as for older children. Examining correlates specific to preschool children is important for the development of appropriate interventions targeting reductions in sedentary behaviour in that age group.

The purpose of the present paper is to review the correlates of preschool children's sedentary behaviour. Based on social ecological models, influences on sedentary behaviour are grouped according to the five domains identified earlier [23]. This review specifically highlights gaps in the existing literature and areas for possible future research.

## Methods

### Search procedure

Literature included in this review was retrieved from three sources. Computerized searches were carried out using Medline, Pubmed, ERIC, Australian Education Index, PsycINFO, Current Contents, Social Science Index, SportsDiscus, Child Development Abstracts, and Health Reference Center - Academic. Manual searches of the reference lists of recovered articles and the authors' extensive personal files were also conducted. Each key term - television viewing; sedentary behaviour; physical inactivity - was searched in conjunction with each term in this group: early childhood; preschool; child; kindergarten, childcare.

An article was included if it: (1) included children aged from three to five years; (2) contained quantitative research and had been published in an English-language, peer-reviewed journal; (3) included a measure of sedentary behaviour as a dependent outcome; and (4) examined associations between explanatory variables and sedentary behaviour. It should be noted that study participants, while referred to as 'preschool children' may not necessarily have attended preschool or childcare at the time they participated in the study.

All measures of sedentary behaviour reported in individual studies have been included to allow for the greatest comparison of findings across studies. Those measures include overall sedentary behaviour (generally measured by accelerometry), television viewing, DVD/video viewing, electronic games, computer use and reading (measured by parental report). However, results have been reported separately for individual behavioural outcomes to determine if correlates vary between behaviours. For cohort or intervention studies, only baseline data were included. A summary of the studies included in this review is presented in additional file 1: Summary of studies investigating correlates of sedentary behaviours.

### Selection of variables

Due to the limited amount of published literature in this area, all variables identified from published studies have been included, irrespective of the number of times they have been investigated across studies. This approach aims to identify the domains which have been explored in the extant literature and to elucidate the multi-dimensional perspective of potential influences on young children's sedentary behaviours. However, where variables were conceptually similar (for instance, one study reported on associations with a number of measures of dietary intake of foods high in energy and low in nutrients), some variables have been combined.

The coding of results follows the model used by Sallis et al. [23] and Hinkley et al. [24]. In that model, the consistency of an association between a correlate and a given sedentary behaviour is determined by the number of reported findings that support the hypothesized association (Table 1). Associations were coded with: 0 (0-33% of studies supporting association); ? (34%-59% of studies supporting an association); or + or - (60%-100% of studies supporting an association). However, given the minimal number of studies which have investigated any individual correlate with a particular behavioural outcome, the strength of the overall association is only reported for a specific behavioural outcome for those correlates which have been investigated in four or more studies. Where four or more studies supported an overall association, the result was coded as ++, - -, or 00 as appropriate.

**Table 1:** Rules for classifying variables regarding strength of evidence of association with sedentary behaviour [23,24]

Studies supporting association (%)	Summary code	Meaning of code
0-33	0	No association
34-59	?	Indeterminate or inconsistent association
60-100	+ -	Positive association Negative association

*Note: *Overall association is only given when four or more studies have investigated an association between a potential correlate and sedentary behaviour.

## Results

Data were collected and analysed between March 2008 and September 2009. Twenty-nine studies were identified, of which one was published in 1993 and the remainder were published between 2002 and 2009. The ages of children in those studies ranged from six months to six years. Only results specific to preschool children (ages 3-5 years) have been included. Methods used for data collection included accelerometry (9 studies [15,16,25-31]), parental checklist (1 study [32]), parental time use diary (1 study [33]), parental survey (11 studies [34-44]), direct observation (5 studies [14,45-48]), parent survey and accelerometry (1 study [49]) and combined heart rate and observation (1 study [50]). Studies largely failed to report reliability and validity results for their measure of sedentary behaviour. Of the 29 studies, six reported validity [15,16,28,31,42,51] and eight reported reliability[14,25,26,45-48,50] of their measure of sedentary behaviour. No study reported both reliability and validity of their measure of sedentary behaviour. Additionally, only one study [46] reported reliability of the measure of correlates used. The majority of the studies (59%), were conducted in the USA[14,15,28-30,34-38,42,43,45-48,50]. Four studies (14%) were conducted in Australia [32,33,40,41], four studies originated in Scotland (14%) [16,25,26,31], and one each of the remaining studies were from Germany [39], New Zealand [49], Greece [44] and Belgium [51].

Of the 29 studies identified and included in this review, 16 had a measure of sedentary behaviour as their primary outcome [14,16,29,34-38,40-44,46,50,51]. Eleven of the remaining studies had physical activity as their primary outcome [15,25,26,28,30-32,45,47-49], and one study each had overweight [39] and overall time use [33] as their primary outcomes. Each of those 13 studies also reported on associations between potential correlates and a measure of sedentary behaviour. Variables were identified across four of the five domains discussed above: demographic and biological; behavioural; social and cultural; and physical environment. No psychological, cognitive or emotional variables were identified.

Across the 29 identified studies, 63 variables had been investigated as potential correlates. Of those variables, 44 (69%) were investigated just once each, five (8%) were investigated twice each, six (10%) were investigated in each of three studies, and eight (13%) were investigated four or more times. Studies investigated a median of three (range 1-15) potential correlates. Sample sizes ranged from 64 to 3141, with a median of 280.

Studies investigated associations with a variety of sedentary behaviours as outcomes. The most commonly investigated sedentary behaviour was television viewing, with 16 studies [32-44,46,49,50] investigating associations with that behaviour. Across those 16 studies, 41 potential correlates were investigated. Fifteen studies investigated associations between 22 potential correlates and overall sedentary behaviour as measured by accelerometry or heart rate monitoring [14-16,25-31,45,47-50]. Other sedentary behaviours included DVD/video viewing (three studies [35,39,49] investigating 7 potential correlates), playing electronic games (four studies [32,35,36,48] examining 7 potential correlates), computer use (five studies [32,35,39,40,49] investigating 19 potential correlates), and reading (one study [35] investigating 1 potential correlates). Tables 2 to 5 summarise associations between potential correlates and each of the sedentary behaviours. The most cogent findings are discussed below.

### Demographic and biological variables

Fifteen demographic and biological variables were investigated across 23 studies, as shown in Table 2. The most frequently assessed demographic correlate, investigated in 13 studies, was child's sex, which was found to have an indeterminate association with sedentary behaviour [15,25,27-29,31,47,49,50] and consistently no association with television viewing [33,37-39,44,49,50]. Indeterminate associations were also found for age [34,35,38,43,44], child's body mass index (BMI) [33,38,41-44], parental education [36-38,43,44] and race [38,39,46,50]. Other correlates had been investigated too infrequently to make a robust judgment regarding overall associations.

**Table 2:** Demographic and biological correlates of sedentary behaviours and direction of association

Correlate variables	Sedentary behaviour*		TV viewing		DVD/videos		Electronic games		Computer use		Reading	
	**Assoc**.	Studies	**Assoc**.	Studies	**Assoc**.	Studies	**Assoc**.	Studies	**Assoc**.	Studies	**Assoc**.	Studies
Child's sex (female)	0	[15, 27-29, 47]	0	[33, 37(5-6y), 38, 44, 49-50]	0	[49]			0	[49]		
	+	[25,31,49-50]	+	[37 (3-4y)]								
*Overall assoc*.	*?*		*00*									
Child's age	+	[15,47]	+	[34-35, 50]	+	[35]	+	[35]	+	[35]	+	[35]
	-	[50]	-	[44]								
	0	[28]	0	[38,43]								
*Overall assoc*.			*?*									
Child BMI			0	[41-44]								
			+	[33, 38]								
*Overall assoc*.			*?*									
Race (non-Caucasian)	0	[15, 47,50]	+	[39 (non-German), 46]								
			0	[50]								
			-	[38]								
*Overall assoc*.			*?*									
Motor skill	0	[30]										
Breast feeding duration			-	[38]								
SEP	0	[26]										
	+	[26 (boys)]										
Parents' age			-	[38]					+	[40 (> 40y)]		
Parental education	0	[15]	-	[38,43]					0	[40]		
			0	[37,44]								
*Overall assoc*.			*?*									
Parents' marital status(not married)			+	[37 (5-6y), 38]					-	[40]		
			0	[37 (3-4y)]								
Parental employment status(full-time)			0	[37]								
Parent retired			0	[44]					0	[40]		
Family income			-	[38]					0	[40]		
Parental BMI			+	[38,43]								
Parents studying									0	[40 (pat)]		
									+	[40 (mat)]		

Abbreviations: mat: maternal; pat: paternal; y: years.

* Sedentary behaviour was defined as: levels 1 & 2 in OSRAP 5 point scale [47]; levels 1 & 2 in CARS 5 point scale [50]; CSA/MTI accelerometry cut-point of <1100 cpm [25,26,31]; ActiGraph age-specific cut-points corresponding to ≤1.4 METs (approx. 100 cpm) [15]; ActiGraph age-specific cut-points between 1456 cpm (3 year olds) and 1596 (5 year olds) [27,28]; ActiGraph cut-point <150 cpm [30]; Actical cut-point of <200 cpm [29,49].

### Behavioural attributes and skills variables

Table 3 summarises the associations between behavioural correlates and each of the sedentary behaviours. Twelve studies investigated a total of 14 behavioural variables. Outdoor playtime was found to have no association with television viewing [33,37,39,42]. The remaining 13 behavioural variables had been investigated too infrequently to determine overall associations.

**Table 3:** Behavioural correlates of sedentary behaviours and direction of association

Correlate variables	Sedentary behaviour*		TV viewing		DVD/videos		Electronic games		Computer use		Reading	
	**Assoc**.	Studies	**Assoc**.	Studies	**Assoc**.	Studies	**Assoc**.	Studies	**Assoc**.	Studies	**Assoc**.	Studies
Physical activity			-	[44,50]					0	[40]		
			0	[43]								
Outdoor playtime	-	[48]	0	[37,39,42]	0	[39]						
			-	[33]								
*Overall assoc*.			*0*									
Child attends swim lessons			-	[32]								
Sedentary behaviour			0	[50]								
Sleep			-	[38]								
Reading			0	[37]					0	[40]		
Drawing									0	[40]		
Computer use			+	[40 (wk)]								
			0	[40 (we)]								
Playing console games									+	[40]		
Playing hand-held games									+	[40 (we)]		
									0	[40 (wk)]		
Energy intake			+	[38,43]								
Consumption energy dense foods			+	[38]								
Consumption skim milk/fruit & vegetables			-	[38]								
Multivitamin use			0	[38]								

Abbreviations: we: weekend; wk: week.

* Sedentary behaviour was defined as: levels 1 & 2 in OSRAP 5 point scale [48].

### Social and cultural variables

Only eight studies reported associations between 12 characteristics of the social environment and sedentary behaviours among young children, as summarised in Table 4. Correlates in this domain focused on parental variables, and included teacher education and training. Although none of the correlates studied had been investigated enough to provide an overall association, television time rules was found to have an inverse association with four of the behavioural outcomes (television viewing, DVD/videos, electronic games and computer use) in three studies [32,37,39].

**Table 4:** Social and cultural correlates of sedentary behaviours and direction of association

Correlate variables	Sedentary behaviour*		TV viewing		DVD/videos		Electronic games		Computer use		Reading	
	**Assoc**.	Studies	**Assoc**.	Studies	**Assoc**.	Studies	**Assoc**.	Studies	**Assoc**.	Studies	**Assoc**.	Studies
Presence of siblings			0	[44]								
Television time rules			-	[32, 37 (5-6y), 39]	-	[39]	-	[39]	-	[39]		
			0	[37 (3-4y)]								
Parents limit TV advertising exposure			-	[32]								
Parental encouragement/discouragement for PA	0	[50]	+	[50]								
Parental perception TV helps			+	[37 (5-6y)]								
			0	[37 (3-4y)]								
Parental perception TV hurts			0	[37]								
Parental role model PA			0	[39]	0	[39]	0	[39]	0	[39]		
Maternal smoking			+	[38]								
Parental TV viewing time			+	[44]								
Parental time with child			0	[44]								
PA training & education (teachers)	-	[14]										
Preschool teacher education (college)	0	[45]										

Abbreviations: y: years.

* Sedentary behaviour was defined as: levels 1 & 2 in OSRAP 5 point scale [14,45]; levels 1 & 2 in CARS 5 point scale [50].

### Physical environmental variables

Correlates investigated in this domain are summarised in Table 5. Primarily, studies focused on potential correlates in the home physical and preschool/childcare centre environments. There were 22 physical environmental correlates examined across 12 studies. Of those 22 correlates, 11 were relevant to the preschool or childcare centre environment. Generally, variables identified in the physical environment were not associated with young children's sedentary behaviours.

**Table 5:** Physical environmental correlates of sedentary behaviours and direction of association

Correlate variables	Sedentary behaviour*		TV viewing		DVD/videos		Electronic games		Computer use		Reading	
	**Assoc**.	Studies	**Assoc**.	Studies	**Assoc**.	Studies	**Assoc**.	Studies	**Assoc**.	Studies	**Assoc**.	Studies
*Home and neighbourhood variables*												
TV in bedroom			0	[36-37]			+	[36]				
Number of TVs in home			0	[39]	0	[39]	0	[39]	0	[39]		
Playstation in home							+	[32]				
Computer in home									-	[32]		
Internet connection in home			-	[32]								
Constant television			+	[32, 37 (3-4y)]								
			0	[37 (5-6y)]								
Backyard characteristics			0	[39]	0	[39]	0	[39]	0	[39]		
Neighbourhood safety			-	[42]								
Region of residence (urban)			+	[44]								
Day of the week (weekday)	-	[27]	0	[42]								
Season		Higher in spring than summer or fall [16]										
*Centre-based variables*												
Attends out-of-home care									0	[40]		
Active opportunities	-	[14]										
Sedentary environment	+	[14]										
Preschool attended		[15] Varies with centre										
Preschool quality	-	[45]										
Preschool field trips	0	[45]										
Community involvement	0	[45]										
Preschool TV/computer time	0	[45]										
Time outdoors at preschool	0	[45]										
Free time at preschool	0	[45]										
Preschool class size	0	[45]										

Abbreviations: y: years.

* Sedentary behaviour was defined as: levels 1 & 2 in OSRAP 5 point scale [14,45]; CSA/MTI accelerometry cut-point of <1100 cpm [16]; ActiGraph age-specific cut-points corresponding to ≤1.4 METs (approx. 100 cpm) [15]; ActiGraph age-specific cut-points between 1456 cpm (3 year olds) and 1596 (5 year olds) [27].

Additional file descriptions text (including details of how to view the file, if it is in a non-standard format).

## Discussion

This review of correlates of preschool children's sedentary behaviours has found that two correlates were consistently unrelated to preschool children's television viewing: outdoor playtime and sex of the child. However, the association between sex and overall sedentary behaviour in preschool children was indeterminate. Further, lack of consistency between studies resulted in an indeterminate association between television viewing and child's age, BMI, race, and parental education. All remaining 57 potential correlates were investigated too infrequently to determine an overall association with any of the behavioural outcomes examined. However, it is worth highlighting the possible association between parental rules and child sedentary behaviours. Although parental rules was investigated in only three studies, its association across behaviours (TV viewing, DVD viewing, electronic game and computer use) was consistently inverse. While this review identified a moderate number of studies investigating a large range of potential correlates of preschool children's sedentary behaviours, consistency of correlates investigated across those studies was lacking. Therefore, it is not possible to draw conclusions about associations in most cases.

These findings are in contrast to the review by Gorely et al. [22] which investigated correlates of television viewing in school-aged children and youth and identified associations for 21 correlates. However, that review included studies for children and youth aged two to 18 years. Although those authors included 10 studies among children aged two to six years, the review did not present findings for those children separately, and therefore direct comparisons with the current review are not possible. However, that review found that ethnicity, body weight, snacking, parents' television viewing, weekend days and having a television in the bedroom were all positively associated with television viewing, while parental income and education, and the number of parents in the home were all negatively associated with television viewing. The current review largely found inconsistent associations between those correlates and children's sedentary behaviour. In addition, Gorely et al. [22] found no association between sex and television viewing which is consistent with the findings of the current review. More recently, van der Horst et al. [21] reviewed correlates of sedentary behaviour in children and youth aged four to 18 years. That review identified several correlates of sedentary behaviour for 13 to 18 year old youth, but identified only four studies which investigated correlates of sedentary behaviour in children aged 4 to 12 years. Similar to the current review, van der Horst et al. [21] concluded that there was insufficient evidence to draw conclusions about potential correlates of sedentary behaviour in children.

### Limitations of the published literature

Most of the studies included in this review were cross-sectional, thereby inhibiting opportunities to clearly identify causality of potential influences on behaviour. The majority of research conducted into correlates of sedentary behaviour has used relatively small (half the studies had fewer than 300 participants) and potentially non-representative samples. Where small samples are used, weak, but potentially meaningful, associations may go undetected. Furthermore, although there were seven studies included in this review which used large samples (> 1000 participants), those studies include the possibility of reporting trivial associations as statistically significant when they may not be meaningful.

There was little consistency between studies in the variables examined within specific settings. For example, several studies investigated the potential influence of variables in the preschool or childcare setting [14,15,45], yet those studies each explored different variables. Influences in a given setting may include physical environmental correlates, such as the layout of the centre itself, and social correlates, such as teacher/staff education or support. While exploring diverse correlates is valuable in terms of covering the breadth of influences on preschool children's sedentary behaviours, examination of the same variables in different studies utilizing different samples is necessary to build a body of evidence to support or refute the potential influence of any individual variable.

Some potentially important variables are under-researched. For instance, potential correlates such as socioeconomic position, growth and maturation of the child (with the exception of age), parental influences, and social and physical environmental influences in preschools and childcare centres as well as other settings (e.g., neighbourhood), are generally overlooked or poorly researched, and may be important influences on sedentary behaviours.

Methodologies in data collection techniques varied across studies. A range of instruments were employed to measure sedentary behaviours and their associated correlates. Those instruments all have different levels of reliability and validity, and may also measure different outcomes (movement, lack of movement, time spent in specific behaviours) thus making it difficult to compare evidence collected across different studies. Most studies did not report validity and reliability data for instruments used to assess sedentary behaviour, and only one study reported reliability for the measures of correlates used [46]. Given this paucity of reliability and validity data, it is not possible to make statistical adjustments for measurement error in a given study.

Many sedentary behaviours, such as television viewing, computer and electronic game use, are difficult to measure in the preschool population, and rely largely on parental proxy-reports, as most preschool children do not have the cognitive ability to self-report [52-55]. Protocols around measurement periods of sedentary behaviours varied greatly, and included estimates of 'average' television viewing time [36], 48 hours of observation and heart rate monitoring [50], and 10 days of accelerometry [28]. Such differences in study protocols further confound comparisons between studies and the representativeness of the behaviour being investigated. Additionally, certain correlates of sedentary behaviours may be difficult to measure in preschool-aged children, particularly psychological, cognitive and emotional constructs, as reflected by the absence of studies investigating such potential influences.

More than half the studies included in this review investigated television viewing as their behavioural outcome, thereby neglecting other, potentially important, sedentary behaviours. Studies investigating positive sedentary behaviours in young children such as reading, drawing, quiet play (e.g. with blocks or dolls, etc), and crafts are unmistakably absent, perhaps because it is perceived that those behaviours are unlikely to be detrimental to children's health. Those behaviours may themselves be related with healthful developmental outcomes, and may also have potentially important correlates which could be targeted in interventions to decrease time in screen-based behaviours such as television viewing and e-game use.

### Future directions

Given the large proportion of time that preschool children spend being sedentary, additional studies are required to further understand the influences on those behaviours. Further, additional evidence would support the development of interventions to decrease the proportion of time preschool children spend in potentially unhealthy sedentary behaviours.

Studies which investigate potential influences across a number of settings or contexts would enhance understanding of the multi-dimensionality of influences on preschool children's sedentary behaviour. The collection of sedentary behaviour data using reliable and valid measures across a range of locations, and at different times during the day would further enhance understanding. Almost half the studies included in this review did not have a measure of sedentary behaviour as their primary outcome or purpose, and the psychometric properties of the sedentary behaviour measures were rarely reported. This again points to the lack of research being undertaken in this area in preschool children. Studies designed to primarily investigate those behaviours in young children are necessary to develop a more robust understanding of their participation in sedentary behaviours and inform future interventions.

## Conclusions

In summary, there is a dearth of literature on the correlates of sedentary behaviours in preschool children. That which does exist provides largely inconclusive evidence of correlates of sedentary behaviours in that population. Although potential correlates have been identified across four of the domains of the social ecological model, consistent evidence exists for only two variables: sex and outdoor playtime, both of which were shown to have no association with television viewing, and sex had no association with overall sedentary behaviour. The factors which influence sedentary behaviours in preschool-aged children are multi-dimensional and complex. Further evidence is necessary to more fully understand which variables may be important in the development of interventions to support healthful outcomes in preschool children.

## Competing interests

The authors declare that they have no competing interests.

## Authors' contributions

TH was involved in acquisition, analysis, and interpretation of data, drafting and manuscript writing. JS was involved in analysis and interpretation of data, drafting and critically revising the manuscript. ADO and ST were involved in the conception and design of the paper and played a role in critically revising and editing the manuscript. All authors read and approved the final manuscript.

## Supplementary Material

Additional file 1 **Summary of studies investigating correlates of sedentary behaviours**. Table. Click here for file
