# Supplementary material for: Correlates of sedentary behaviours in preschool children: a review
Source: Int J Behav Nutr Phys Act. 2010 Sep 8;7:66. doi: 10.1186/1479-5868-7-66 (PMC2945987; doi:10.1186/1479-5868-7-66)
Supplement: Additional file 1 — Summary of studies investigating correlates of sedentary behaviours. Table. [file 1479-5868-7-66-S1.DOC]

**Additional file 1 -** Summary of studies investigating correlates of sedentary behaviours

| **Study** | **Design, purpose, analyses, and correlates investigated** | **Country** | **Sample characteristics** | **Measure (s) of SB** | **Measurement period** | **Validity/reliability of SB measure** | **Reported SB level** |
| --- | --- | --- | --- | --- | --- | --- | --- |
| Bower et al., 2008 [14] | Cross-sectional survey to determine which aspects of the childcare environment were associated with children’s PA and SB  Analyses: descriptive, correlations, ANOVA, post-hoc analyses, ANCOVA, regression  Correlates: active & sedentary opportunities, sedentary environment, portable play environment, fixed play environment, staff behaviours, PA training & education, PA policy | USA | 20 childcare centres; average racial/ethnic distribution reported by centre directors was 33% black, 59% white, 4% Hispanic, and 4% other. Children aged 3-5y. | Direct observation (OSRAP), 15s observe/15s record cycles | 4 randomly selected children in each centre observed for 8 x 32min periods over 2 consecutive d | Intraclass correlations >0.90, % agreement – 75%-99%. | µ=55.35% (SD=8.50%) of observations in SB |
| Pate et al., 2004 [15] | Cross-sectional study to describe PA levels of preschool children, identify demographic variables and determine variation among preschools.  Analysis: ANOVA multiple linear regression, two-step regression analysis.  Correlates: age, ethnicity, sex, parent education, preschool attended. | USA | n=247; 115M, 132F; 3-5y; 65% black, mean BMI 16.1(SD1.8) | Accelerometry ActiGraph 7164, 15 s epoch, Sirard et al. [57] cut-points | µ=4.4h/d (SD=1.3h, range 0.4-7.8h) for µ=6.6d (SD=2.3, range 1-11d); minimal inclusion criteria ≥1h on ≥3d | Validity previously reported. Reliability not reported. | µ=42.1(SD=5.8) min/h |
| Fisher et al., 2005 [16] | Cross-sectional study examining seasonal variation in objectively measured habitual PA & SB.  Analyses: Kruskall-Wallis test, Mann-Whitney U tests, ANOVA, t tests, regression.  Correlates: season | Scotland | n=209; µ=4.8y (SD = 1.2y); 101M, 108F. | Accelerometry CSA/MTI 7164 Puyau et al. [58] and Reilly et al. [59] cut-points, epoch not reported | µ=56.6h (SD=30.0, range 18.0 to 217.3h). 3d: 2 wk, 1 we d for 3yo; 7d for 5yo. ≥6h wear time. | Validity previously reported. Reliability not reported | SB cut-point <1100cpm. Spring: µ=79.5% (62.1%-93.0%) of time; Summer: µ=74.2% (61.0%-88.4%) of time; Fall: µ=76.1% (54.7%-87.4%) of time; Winter: µ=76.6% (59.3%-91.9%) of time in SB. |
| Montgomery et al., 2004 [25] | Cross-sectional study assessing relations between total EE and PAL during different intensity activities.  Analyses: correlation, multiple regression.  Correlates: sex, physical activity level (PAL). | Scotland | n=104; 52M, 52F; µ=5.4y; 36 in preschool, 68 in school | Accelerometry CSA/MTI uniaxial  Accelerometer, 1 min epochs, Puyau et al. [58] and Reilly et al. [59] cut-points | Waking hours, 3d for preschool, 7-10d for primary, median recording was 30.3h in preschool and 78.3h in school chn, 6-13 waking h/d | Reliability previously reported. Validity not reported | % time in SB: M=73% (61-90%), F=79% (63-93%). |
| Kelly et al., 2006 [26] | Cross-sectional study to test hypothesis that habitual PA is associated with SEP.  Analyses: ANOVA, ANCOVA, backward stepwise multivariate model.  Correlates: sex, age, BMI, SEP. | Scotland | n=339; µ=4.2y (SD = 0.5y) | Accelerometry, 1 min epochs, Reilly et al. [59] and Puyau et al. [58] cut-points | 6 d, µ=54.9h, SD13.8h | Previously observed negligible day-to-day variation and no systematic within-child, within-day variation in accelerometry output | 77% of time sedentary (<1100 cpm) |
| Cardon et al., 2009 [27] | Cross-sectional study examining PA and SB levels in 4- and 5-year-old children.  Analyses: t-tests, ANOVA.  Correlates: day of week, sex. | Belgium | N=76; age 4-5y, 37M, 39F. | ActiGraph 7164 accelerometer; Sirard [60] cut-points, 15s epoch | 4d, 2 wk, 2 we days | Validity previously reported; reliability not reported. | Total: 84.7% of wear time; 84.9% and 84.6% on wk and we respectively; boys 546 and 645 min/d on wk and we d respectively; girls 619 & 593 min/d on wk and we d respectively. |
| Hannon et al, 2008 [28] | RCT to increase preschoolers’ PA intensities.  Analyses: descriptive, general linear models.  Correlates: sex, age. | USA | N=64; 30M, 34F; 3-5y; mostly Caucasian | ActiGraph GT1M accelerometer; 15s epochs; Sirard [60] cut-points | 10d | Validity previously reported; reliability not reported | Boys: 56.9-54.1%; girls 59.6-56.7% time in SB. |
| Temple et al., 2009 [29] | Cross-sectional study investigating SB and PA in family child care.  Analyses: ANOVA.  Correlates: sex. | USA | N=65; 49% girls, 3-5y | Actical accelerometers, 15s epoch; Pfeifer et al [61] cut-points | 1-4 d; mean total wear time 16.63h (SD=8.46), range 5.11-32.44h; median 14.47) | NR | Girls: median 39.88, mean 40.047, SD=4.44; boys median 39.80, mean 38.97, SD=4.56 min/h sedentary. |
| Williams et al., 2008 [30] | Cross sectional study to examine the relationship between motor skill performance and physical activity in preschool children.  Analyses: Pearson correlations, descriptive.  Correlates: motor skill performance scores. | USA | N=198; 80 3y, 118 4y | ActiGraph 7164 accelerometers; 15s epochs; Pate et al. cut-points [62] | 8-10 d at preschool and 2 we d at home; up to 5d wk data and 2d we data used. | NR | 55% of wear time sedentary |
| Fisher et al., 2005 [31] | Cross-sectional study assessing associations between PA and FMS.  Analyses: correlation, Kruskal-wallis tests, Mann-Whitney tests.  Correlates: sex | USA | N=394, 3-5y, µ=4.2 (SD=0.5, range 3.6-5.0)y | CSA/MTI 7164 accelerometer, Puyau et al. [58] and Reilly et al. [59] cut-points, epoch not reported. | ≥9h/d for ≥3d to maximum of 6d | Validity previously reported. Reliability not reported. | Time inactive µ=76.3% (SD=6.8, range 53.6 to 92.9, median 77.2) of time worn. |
| Spurrier et al., 2008 [32] | Cross-sectional study to assess associations between characteristics of the home environment and preschool children’s PA and dietary patterns.  Analyses: descriptive, ANOVA, post hoc, correlation.  Correlates: parental role-model of PA, backyard characteristics | Australia | N=280; µ=4.8y (SD = 0.21y, range 4.1-5.4y); 50% M | 12 item parent report checklist for outdoor play and small screen entertainment | Once | NR | Mean small screen entertainment score range 0-11, possible range 0-24. |
| Tey et al., 2007 [33] | Cross-sectional study to explore time-use patterns in children aged 5 y.  Analyses: descriptive; t-tests, Mann-Whitney U tests.  Correlates: BMI; sex; time outdoors. | Australia | N=84; 43% male; mean age 5.1y (SD=0.1). | Parent completion of light time use diary | 2 wk & 1 we d | NR | TV viewing: median 1h 54 min/d. |
| Dennison et al., 2002 [34] | Cross-sectional study to describe the TV/video viewing habits of a multiethnic, low-income preschool population of children and to determine whether TV/video viewing is related to their adiposity  Analyses: t-test, ANOVA, Scheffe post-hoc tests, chi-square, multiple logistic regression  Correlates: age | USA | N=2,761 low income parents with children aged >1 through <5 y | Parent survey – usual TV/video viewing time on wk and we d | Once | Not validated. Reliability not reported. | 96.4% (3y) - 97.7% (4y) watch TV; 16.3 (3y)-18.4 (4y) mean h/wk; 47.7(3y)-57.1% (4y) watch >2 h/d. |
| Anand et al., 2005 [35] | Cross-sectional study examining media-use behaviours of young children.  Analyses: bivariate regression, multivariate regression.  Correlates: age | USA | N=1065 parents; chn age 6m – 6y | Parent report on television viewing, DVD/video viewing, reading/being read to, playing X-box type games, computer games, other computer use. | 1d either the day before the interview or a typical day. | NR | NR |
| Dennison et al., 2004 [36] | RCT to decrease TV viewing time  Analyses: correlation  Correlates: TV in bedroom | USA | N= 77, mean age 3.9y (SD 0.07) IV and 4.0y (SD 0.01) CON, range 2.5-5.5y, attend preschool or day care | Parent survey reporting on TV viewing time | Once – estimate of average wk in previous week and estimates of we day | NR | TV/video viewing = 12.8-13.5h/w (SD1.0-1.3) for IV & CON respectively; video/computer game = 2.6-1.5 (SD0.5-0.5) for IV & CON respectively |
| Vandewater et al., 2007 [37] | Cross-sectional study to describe media access and use among children aged from birth to six years.  Analyses: descriptive, logistic regression, multivariate analyses of covariance  Correlates: availability of TV, cable/satellite, number of TVs, portable DVDs, VCR/DVD player, video game consoles, handheld video games, computers, internet access, TV in child’s bedroom; use of and amount of time using TV, video/DVD, video games, computer games, other computer, electronic books, music; ethnicity, education level, employment; reading, time outdoors | USA | N=1045, 0-6y; 60% non-Hispanic white, 14% black, 20% Hispanic/Latino, 6% other. | Parent telephone survey of media ownership, access and use on the previous day | Once | NR | 82% of 3-4 y and 78% of 5-6 y watched TV on previous day; mean TV viewing time was 89.67 (SD=57.97) and 73.99 (SD=53.45) min for 3-4y and 5-6y respectively; 56% of 3-4y and 70% of 5-6y met AAP guideline of ≤2h/d of TV |
| Miller et al., 2008 [38] | Cross-sectional study to examine the association between TV/video viewing & markers of diet quality among children aged 3y  Analyses: descriptive, bivariate analyses, multiple linear regression  Correlates: sex, age, BMI, parent age, parent education, parent marital status, family income, race, parental BMI, sleep, energy intake, consumption energy dense foods, consumption skim milk/fruit & vegetables, multivitamin use, maternal smoking | USA | N=613 M & 590 F age 3y, mean age 3.2y (SD=0.2) | Proxy report survey– no. of hours child watch TV on ave wk & we in past month | Once | NR | Mean TV viewing time was 1.7 h/d |
| Kuepper-Nybelen et al., 2005 [39] | Cross-sectional study investigating prevalence of overweight and its correlates according to nationality.  Analyses: multiple logistic regression.  Correlates: nationality, overweight, day of week. | Germany | n=1979; 21.1% 5y, 78.9% 6y, recruited at obligatory health examination before school entry. | Proxy-report survey of TV viewing on wk & we | Once | NR | % watching >2h TV/d: German – 8.6% wk, 19.0% we d; Other nationality – 31.9% wk, 47.2% we d. |
| Straker et al., 2006 [40] | Cross-sectional study to describe the exposure of young children to various forms of ICTs  Analyses: Wilcoxon Signed Rank test, logistic regression  Correlates: watching TV/videos, playing console games, playing hand-held electronic games, reading, drawing, PA, parents’ age, household income, out of home care, parents’ education, parents’ studying, parents’ employment, parents’ marital status | Australia | N=1600, 5y | Parent survey of TV/video viewing, playing e-games, computer use, reading or looking at books, and drawing, painting or writing on paper | Once; four point scale of participation in specified activities on wk & wd | NR | 56% of chn used computers each week; 28% used console games; 9% used hand-held games; 12.8% and 18.9% of chn watched >2h/d of TV on wk & we d respectively |
| Okely et al., 2009 [41] | Cross-sectional study to determine compliance with NASPE guidelines for PA and AAP recommendations for TV viewing in preschool children.  Analyses: PROC MIXED, descriptive.  Correlates: sex, weight status, wk vs we d | Australia | 11 centres, n=386 children; 201 boys, 185 girls, µ=3.96y (SD = 0.76y). | Parent report – h in TV viewing for normal wk & we d | Once | NR | µ=2.1h (SD = 0.1h) watching TV. |
| Burdette et al., 2005 [42] | Cross-sectional survey to test hypothesis that obese children spend less time playing outdoors and more time watching TV when in unsafe neighbourhoods  Analyses: ANOVA, chi-square, linear models, bivariate analyses  Correlates: neighbourhood safety | USA | n=3141; 1665M, 1476F, µ=3.25y (SD = 0.25y); 35% in households below poverty line; 50% non-Hispanic black; 25% non-Hispanic white; 25% Hispanic; 18% obese | Parent survey reporting TV/video viewing on typical wk & we d | Parental report of the number of hours their child ‘typically’ spends outdoors each wk and each we d. | Previously shown to correlate with PAL as measured by accelerometer | TV viewing: wk - µ=190 (SD=136) min; we - µ=191 (SD=152) min |
| Proctor et al., 2003 [43] | Cohort study examining TV viewing and change in body fat from preschool to early adolescence.  Analyses: descriptive  Correlates: age; activity level; anthropometry; energy intake; parental BMI; parental education; parental activity. | USA | N=106; mean age 4.0y. | Parent report of ave amount of TV viewing each day | Once | NR | Tertiles: lowest 1.1±05; middle 1.6±0.7; highest 2.4±1.6 h/d of TV viewing. |
| Kourlaba et al., 2009 [44] | Cross-sectional study examining television viewing time of preschoolers and associated factors.  Analyses: descriptive, contingency tables, chi-square, Mann-Whitney or Kruskal-Wallis tests, Bonferroni, Spearman correlation, multiple logistic regression, discriminant function analysis  Correlates: PA, weight, recumbent length, standing height, BMI, parent education, parent age at birth of child, birth order, siblings, residence, maternal employment, time parents spent with child we & we d. | Greece | N=2374, age 1-5y, | Parental report of TV viewing time | Once reporting usual wk and we | NR | Mean TV viewing time = 1.32 (SD 1.12) h/d (1 h & 19 min/d) |
| Dowda et al., 2004 [45] | Cross-sectional study to determine the influence of preschool policies/practices, and overall quality of preschools, on MVPA in children aged 3-5 y  Analyses: mixed model ANOVA  Correlates: type of preschool, teacher education, no. of field trips, preschool quality, preschool time outdoors class size, community support, computer/TV use at preschool | USA | n=266; 126M, 140F. ≥6h/d, 5d/w at the preschool; 3-5y | Direct observation (OSRAP) 15s observe/15s record cycles | 1h on 2 to 3d | ICC coefficients for mean activity rating ranged from 0.91 to 0.98. Percent agreement for five activity categories ranged from 75% to 99%. | No overall SB level reported. |
| Sallis et al., 1993 [46] | Cross-sectional study examining correlates of children’s PA and SB.  Analyses: correlations, regression.  Correlates: ethnicity. | USA | n=347; µ=4.4y (SD = 0.5y); 201 Mexican-American, 146 Anglo-American | Direct observation (BEACES) coded 1/min | 4x1h in home evening visit, 30 min prior to evening meal | Interobserver agreement above 90%. Reliability reported for many measures. | TV viewing: level NR |
| Pate et al., 2008 [47] | Cross-sectional study to investigate correlates of physical activity in children attending preschools.  Analyses: ANOVA, regression.  Correlates: age, sex, race. | USA | N=493, 3-5y; 51% males; 55% African American | Direct observation (OSRAC-P), 5s observe, 25s record, 2 obs/ min | 10-12 30 min sessions /child | IOA range 83-100%, mean 91%; Kappa 0.8-0.95; mean 0.82. Validity not reported | 83.4% sedentary |
| Brown et al., 2009 [48] | Cross-sectional study describing PA of young children and environmental events associated with those behaviours.  Analyses: descriptive, logistic regression  Correlates: indoor vs outdoor | USA | N=476, 3-5y | Direct observation (OSRAC-P); 5s observe, 25s record, 2 obs/min for 60 intervals/session | 30 min sessions; indoor obs: mean of 327.5 min/child (SD 29.5min); outdoor obs: mean 34 min/child (SD 24.5 min) | IOA 80%; kappa coefficient means ranged from .82 to .99, except for adult & peer prompts which was .27 | 94% of intervals sedentary during inside periods (87% of the time); 56% of intervals sedentary during outside periods (10% of the time); overall, 89% of time sedentary |
| Taylor et al., 2009 [49] | Cohort study investigating patterns and changes in PA.  Analyses: random coefficient models, descriptive.  Correlates: sex. | New Zealand | N=244; 44% F; 3y at baseline; 87% Caucasian; 10.8% Maori, 3.7% Pacific Islanders | Parent report survey; Mini-Mitter Actical accelerometers; Pfeiffer et al [61]cut-points. Epoch length not reported. | Survey once with report of time spent each wk in activities. Monitor for 5 consecutive d. | NR | Girls: computer 2 (5), TV 54 (43), VHS/DVD 29 (25), total screen time 86 (59), total sedentary time 193 (83) min/d.  Boys: computer 3 (7), TV 51 (39), VHS/DVD 32 (31), total screen time 86 (61), total sedentary time 170 (97) min/d. |
| Jago et al., 2005 [50] | Cohort study examining how variables were associated with PA and SB in a tri-ethnic cohort.  Analyses: descriptive, ANOVA, post-hoc analyses using paired t tests, Pearson correlations, linear-regression.  Correlates: TV viewing, age, physical activity, ethnicity, sex, TV viewing, SB, parental encouragement, parental discouragement. | USA | n=149; 73M, 76F; µ=4.4y (SD = 0.6y); 37% African-American, 37% Anglo-American, 26% Hispanic | Direct observation (CARS, activity levels recorded at the start of each min and then any changes during the min); Quantum XL telemetry HRM | 6-12 hrs of obs/d at same time as heart rate monitoring (4d) | HRM reliability previously reported. Validity for HRM not reported. Validity & reliability for observation not reported. | At baseline SB– µ=52.9 (SD=17.6) min/h; TV viewing – µ=9.7 (SD=8.2) min/h |

Abbreviations: ave: average; BEACHES: Behaviours of Eating and Activity for Child Health Evaluation System; BMI: body mass index; CARS: Children’s Activity Rating Scale; CON: control group; d: day; DVD: digital video disc; EE: energy expenditure; F: female; FMS: fundamental movement skills; h: hours; HRM: heart rate monitoring; ICC: intraclass correlation; IOA: interobserver agreement; IV: intervention group; M: male; m: month; min: minute; MVPA: moderate to vigorous physical activity; NR: not reported; obs: observations; OSRAC-P: Observational System for Recording Physical Activity in Children – Preschool Version; OSRAP: the Observation System for Recording Activity in Preschools; PA: physical activity; PAL: physical activity level; s: second; SB: sedentary behaviour; SEP: socioeconomic position TV: television; VCR: video cassette recorder; we: weekend day; wk: weekday; y: year.
